# Cryopreservation Protocols and the Associated Ultrastructural Changes in Dormant Buds of *Vitis amurensis*

**DOI:** 10.3390/plants13243590

**Published:** 2024-12-23

**Authors:** Dan Sun, Peijin Ni, Jian Liu, Zhenxing Wang, Guangli Shi, Meng Li, Xuanhe Zhang, Jun Ai

**Affiliations:** College of Horticulture, Jilin Agricultural University, Changchun 130118, China; sundan@jlau.edu.cn (D.S.); zhenxinghd@aliyun.com (Z.W.);

**Keywords:** conservation of resources, dormant buds, viability, *Vitis amurensis*, structural changes

## Abstract

There is an urgent need for the cryopreservation of dormant buds to conserve the genetic resources of woody plants, particularly fruit trees, as this method is less time-consuming and relatively inexpensive. In the present study, three different cryopreservation protocols were tested on dormant buds from three varieties of *Vitis amurensis* Rupr. The explants were collected between November 2017 and March 2018. Twig segments harvested from field-grown plants, each containing one dormant bud, were desiccated in a low-temperature test chamber at −5 °C. The viability of the buds was highest (45%) after 28–30 days of desiccation, when the moisture content was approximately 25–30%. Cryopreservation using the CP3 protocol (which involves decreasing the temperature at a rate of 0.1 °C/min to −30 °C and holding this temperature for 24 h, followed by a 0.5 °C/min decline to −80 °C, a 1 °C/min decline to −180 °C, and finally reaching −196 °C in a CryoMed controlled rate freezer) significantly enhanced the viability (66.67%) when the samples were packed in aluminum-foil bags. Additionally, immersing the twigs in ice-cold (4 °C) water for 24 h in a refrigerator during thawing proved to be more conducive to viability. The dormant buds of all three *V. amurensis* varieties collected in January exhibited the highest viability after cryopreservation, followed by those collected in February and December. In contrast, the dormant buds collected in November and March showed the lowest viability after cryopreservation. The average viability of twigs of ‘Shuanghong’, ‘Zuoshanyi’, and ‘Shuangfeng’ collected between 2019 and 2021 all exceeded 60%. After the cryopreservation process, the outer multilayered cells in the buds were completely damaged; however, the inner cells exhibited moderate damage and were able to resume growth after thawing. Therefore, based on graft viability and histological observations, the dormant bud cryopreservation protocols tested in this study could be applicable to these three *V. amurensis* varieties.

## 1. Introduction

The conservation and availability of a wide variety of fruit germplasm including varieties and wild relatives require a strong genetic resource pool. Traditionally, field gene pools serve as ex situ resources for problematic materials. However, several limitations that hinder their utility pose significant threats to germplasm security [1]. Genetic resources are vulnerable to pests, diseases, natural disasters such as drought, human error, and vandalism. Furthermore, germplasm exchange under these conditions carries substantial risks due to the potential transfer of diseases through vegetative propagation [1,2]. Current in vitro gene pools offer a viable option for the short-term and medium-term preservation of various crops [3] including temperate fruit trees and horticultural species [4,5,6,7]. Nevertheless, these in vitro gene pools are not ideal for long-term preservation, as this method can be costly and may result in resource loss due to contamination or technical challenges during regrowth intervals.

Cryopreservation is a valuable tool for the long-term storage of clonally propagated plants and seeds [8,9]. Significant advancements in plant cryopreservation have occurred since the initial experiments conducted from the 1970s to the 1990s, leading to the development of various cryopreservation techniques since the 2000s. Numerous studies on germplasm cryopreservation have been published for a wide range of plant species including herbs, vines, shrubs, and trees across diverse growth regions, from tropical and subtropical to temperate climates [8,10]. Propagules such as shoot tips and dormant buds are candidates for clonal cryopreservation, in which the traits of interest from the parent plant are retained [11,12]. The cryopreservation of dormant buds (DBs) is less time-consuming than that of the shoot tips, as DBs do not require aseptic culture or additional time for excision, increasing the cryopreservation capacity [13]. In a pioneering study, Sakai initiated the cryopreservation dormant buds on *Salix* and *Populus*, and then extended this to other species such as *Vitis* [14,15,16,17]. Since then, dormant bud cryopreservation has been used for other species such as *Betula pendula* [18], *Prunus cerasus* [19], blueberry [20], *Malus* [21], *Ulmus* [22], *Fraxinus* [23], Pyrus [24], *Pinus sylvestris* [25], and other tree species. Although there may have been successful scientific reports, the dormant bud approach has not been widely adopted for the preservation of crop germplasm. One possible reason for this is that the method is primarily utilized for shrubs and trees that can survive dormancy; however, the DB of crops may not yield satisfactory post-cryopreservation viability.

*Vitis amurensis* Rupr. (Vitaceae), a nature-state vine, is widely distributed from the northeastern regions of China to northern Korea. It is considered as one of the most desirable wine grape varieties in China [26]. Modern pharmacological studies indicate that *V. amurensis* exhibits anti-inflammatory [27], antimicrobial [28], antioxidant [29,30], and anticancer properties [31,32]. *V. amurensis* vines are generally dioecious, although hermaphroditic vines have also been occasionally reported. This plant species demonstrates high resistance to cold temperatures [33], allowing the viability of branches and roots at −40 °C and −16 °C, respectively [34]. Consequently, *V. amurensis* is regarded as one of the most valuable germplasm resources in grape breeding. The cryopreservation of *Vitis* shoot tips and dormant buds has been reported in some species, but most studies have been carried out on shoot tips, and only one study has been published on dormant buds [35,36,37,38]. Limited results were obtained when *Vitis* dormant buds were cryopreserved. To the best of our knowledge, there are currently no reports on the cryopreservation of *V. amurensis* germplasm resources. The cryopreservation of DB is more cost-effective, and the recovery process is simpler, utilizing direct grafting or tissue culture [39,40]. In this study, we investigated the optimal conditions for the cryopreservation of DB of *V. amurensis*. We tested various cryopreservation protocols on three varieties of *V. amurensis* and examined the ultrastructural changes during the successive steps of desiccation prior to cryopreservation as well as after thawing. The findings of this study provide effective guidelines, protocols, and methods for the conservation and sustainable utilization of *V. amurensis*.

## 2. Materials and Methods

### 2.1. Plant Material

**Figure 1:**
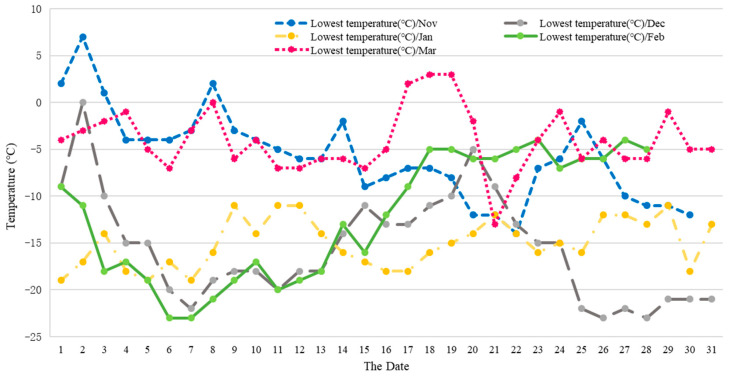
The lowest temperature profile at the orchard of the Jilin Agricultural University (latitude 43°86′; longitude 125°28′), Changchun, China from November 2017 to March 2018.

**Figure 2:**
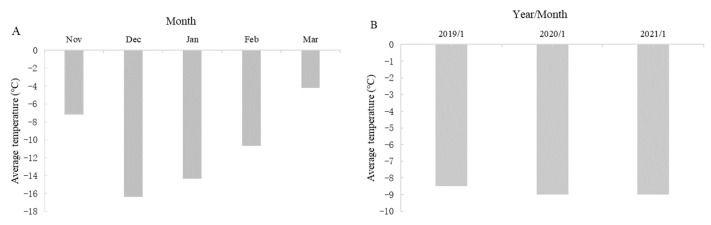
The average temperature at the orchard of the Jilin Agricultural University (latitude 43°86′; longitude 125°28′), Changchun, China. (**A**) The average temperature from November 2017 to March 2018. (**B**) The average temperature of January in 2019, 2020, and 2021.

A total of 1800 one-year-old dormant twigs from 30 individual plants of *V. amurensis* ‘Shuanghong’, ‘Zuoshanyi’, and ‘Shuangfeng’ (planted in 2010) were collected between November (autumn) 2017 and March (spring) 2018, and in January (winter) 2019, 2020, and 2021 at the orchard of the Jilin Agricultural University (latitude 43°86′; longitude 125°28′), Changchun, China. The lowest and average temperatures are shown in Figure 1 and Figure 2. Twigs were cut into 70 mm segments, and each segment contained one dormant bud, which was 1 cm away from the top. The basal and uppermost part of the shoots were not used. The top diameter of the twigs of between 6 and 7 mm was used.

**Figure 3:**
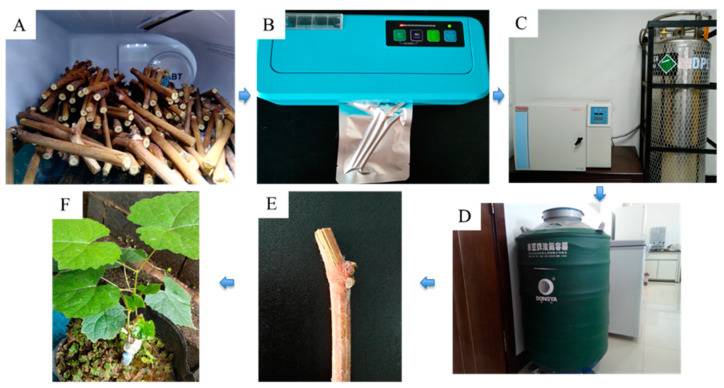
Cryopreservation process of the dormant twigs of *V. amurensis.* (**A**) Twig desiccation at 5 °C in a refrigerator; (**B**) desiccated twigs were vacuumed packed with aluminum-foil bag; (**C**) desiccated twigs frozen in a CryoMed controlled rate freezer; (**D**) desiccated twigs cryopreserved in LN; (**E**) cryopreserved twigs after thawing; (**F**) twigs that survived after grafting for two months.

The standard cryopreservation procedure was as follows (Figure 3). The twigs were desiccated in a low-temperature test chamber at −5 °C for 28 days to the moisture content of approximately 25–30%. Three twigs were packed in aluminum-foil bags and then transferred to a CryoMed controlled rate freezer for cooling down at a rate of 0.1 °C/min to −30 °C, maintaining this temperature for 24 h, followed by a decline of 0.5 °C/min to −80 °C, then a decline of 1 °C/min to −180 °C, and finally dropped into LN. The thawing procedure consisted of immersing the twigs in ice-cold (4 °C) water for 24 h. Twig segments were then grafted onto the rootstock Beta “Zaohong” (1~2-year-old) in a greenhouse maintained at 25 ± 3 °C and 75–95% relative humidity. Rootstocks were selected for grafting based on their thickness, which should match the thickness of the scion. The rootstock was cut flat approximately 5 cm from the rhizome, and the bud eye was removed. A rootstock was split about 1 cm from the center of the stem. The lower part of the scion was split into two opposing inclined planes, each about 1 cm in length. The cut end of the scion was inserted into the incision made in the rootstock, ensuring proper alignment. Finally, the joined sections were secured with thin plastic strips. The buds’ viability was evaluated as the number of buds that sprouted five weeks after grafting.

### 2.2. The Effect of the Desiccation Procedure

Twigs of ‘Shuanghong’ were desiccated in a low-temperature test chamber (DW-100, Yashilin) at −5 °C for 7, 14, 20, 25, 28, 30, or 32 d. Twigs without desiccation were used as the control. Water loss during desiccation and bud viability were determined at each time point. To construct a desiccation curve, the twigs were weighed immediately after desiccation and after drying at 60 °C until reaching a constant weight. The moisture content (MC) was calculated as follows:

MC=(FW−DW)/FW ∗ 100%

Desiccated twigs were packed with aluminum-foil bags and immersed in liquid nitrogen (LN) for 28 d. To thaw, the twigs were removed from the aluminum-foil bags and immersed in ice-cold water (4 °C) for 24 h. The viability of the buds was determined as indicated in Section 2.1. Each treatment contained 30 twigs, and all experiments and analyses were performed in triplicate.

### 2.3. The Effect of Packing and Cryopreservation Procedure

Desiccation was performed as described above, and the amount of water loss and corresponding bud viability were determined at 28 d. Subsequently, the desiccated twigs of ‘Shuanghong’ were either sealed in cryovials (Nunc™, Thermo Fisher Scientific^®^, Waltham, MA, USA) or vacuum sealed in aluminum-foil bags; desiccated twigs subjected to no packing were used as controls. The twig samples were then treated with a different cryopreservation procedure—CP1, CP2, or CP3. For CP1, twigs were immersed in LN directly; for CP2, twigs were exposed to a decreasing temperature gradient of −10 °C for 6 h, −20 °C for 6 h, and −30 °C for 6 h in a low-temperature test chamber (DW-100, Yashilin, Beijing, China), −80°C in an ultra-low temperature freezer (DW-861626, Haier, Qingdao, China) for 6 hm and transferred to LN thereafter; for CP3, twigs were cooled down at a rate of 0.1 °C/min to −30 °C for 24 h, followed by a 0.5 °C/min decline to −80 °C and a 1 °C/min decline to −180 °C in a CryoMed controlled rate freezer (Forma7451TF, Thermo Forma, Waltham, MA, USA). Then, the twigs were submerged in LN for 28 d. To thaw, the twigs were removed from the aluminum-foil bags and immersed in ice-cold water (4 °C) for 24 h. The viability of the buds was determined as indicated in Section 2.1.

### 2.4. The Effect of Thawing and Regrowth Procedures

The moisture content of the twigs was measured at approximately 28–30%. Desiccated twigs were packed in aluminum-foil bags and subsequently cryopreserved using the CP3 procedure described in Section 2.3. These twigs of the ‘Shuanghong’ variety were stored in LN for a duration of 28 d, as previously described. Following this storage period, the twigs were thawed using one of three methods: rapid thawing (10 min immersion in a water bath at 40 °C), room temperature thawing (30 min immersion in water at 25 °C), or slow thawing (24 h immersion in water at 4 °C in a refrigerator). During the slow thawing process, the water was replaced every hour for the initial 6 h. The thawed twigs were subsequently utilized for grafting purposes. The viability of the buds was determined as indicated in Section 2.1.

### 2.5. The Effect of Varieties and Harvest Time

Cryopreservation was conducted using buds from various cultivars, specifically ‘Shuanghong’, ‘Zuoshanyi’, and ‘Shuangfeng’, which were collected during the months of November, December, January, February, and March. The experimental procedures commenced in November and extended through to March of the following year. Desiccation of the twigs was achieved by subjecting them to a low temperature test at −5 °C. The desiccated twigs were subsequently vacuum packed in aluminum-foil bags and frozen according to the CP3 protocol. Following this, the twigs were stored in LN for a duration of 28 d. Thawing was performed slowly by immersing the twigs in water for 24 h at a temperature of 4 °C. The viability of the buds was determined as indicated in Section 2.1.

### 2.6. Sample Preparation for Transmission Electron Microscopy (TEM)

To assess the effects of cryopreservation on the cell and organelle structure, the ultrastructure of cells in the apical meristem was observed under a transmission electron microscope. An assessment was made for the injury degree and seriousness in the duration of the desiccation–freezing–thawing cycle. Frozen buds (1–3 mm^3^) stored in LN for 28 d were sampled under sterile conditions and fixed in 4% glutaraldehyde by vacuum infiltration. The fixed buds were incubated at 5 °C overnight and then rinsed with 0.1 mol/L phosphate buffer (pH 6.8) five times, once each for 5, 10, 15, 20, and 30 min. Subsequently, the buds were fixed in 1% osmium acid at 4 °C for 2 h and rinsed 4–5 times with 0.1 mol/L phosphate buffer (pH 6.8) for 10 min each. Then, the buds were subjected to gradient ethanol dehydration as follows: 30% for 15 min, 50% 15 min, 70% ethanol for 15 min, 80% for 20 min, 90% for 20 min, and 100% for 30 min. Finally, the buds were dehydrated with 100% acetone twice, each for 30 min. The dehydrated buds were penetrated with acetone:embedding agent (Spon812) (3:1) for 2–3 h, acetone:embedding agent (1:1) for 4–6 h, acetone:embedding agent (3:1) for 12 h, and twice with pure embedding agent for 24 h each. The embedding buds were oven dried at 30 °C for 12–24 h and then at 60 °C for 24–48 h. After sample preparation, the embedding blocks were restored and positioned with half-thin parts. Samples were double stained with uranium acetate and lead citrate, and the structural changes in the cells and organelles were observed and photographed by a JEM—1400 TEM.

### 2.7. Experimental Design and Data Analyses

Each treatment contained 30 twigs, and all experiments and analyses were performed in triplicate. An analysis of variance and comparison of means were carried out using SPSS 22.0 (SPSS Inc. Chicago, IL, USA), and significant differences were determined at *p* < 0.05 using Duncan’s multiple range test.

## 3. Results

### 3.1. Effects of Drying Duration and Moisture Content on the Viability of V. amurensis Buds

**Figure 4:**
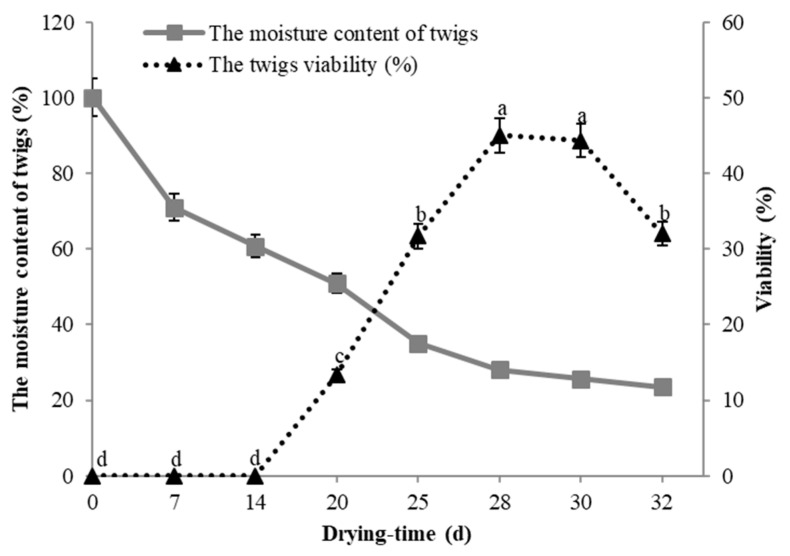
Effects of different drying times on the moisture content in twigs of ‘Shuanghong’ collected in January 2018 and the viability of the DBs after the cryopreservation of *V. amurensis.* Note: Mean values in each point with the same letter were not significantly different at *p* < 0.05 by the Duncan’s multiple range test.

The moisture content of plant tissues is a critical factor that influences their viability following cryopreservation. Figure 4 demonstrates that after 14 days of dehydration at −5 °C, the moisture content of the twigs exceeded 60%, resulting in a viability rate of 0% for the DBs post-cryopreservation. After 20 days of dehydration, the moisture content of the twigs decreased to 50%, and the viability of the DBs after cryopreservation was recorded at 13.33%. As the moisture content decreased, the viability of the buds post-cryopreservation increased. The highest bud viability, at 45%, was observed within a moisture content range of 25–30%, with an average moisture content of 28%. However, with further reductions in moisture content, the viability of the DBs also declined.

### 3.2. Effects of Packing and Cryopreservation Protocols on the Viability of V. amurensis Buds

The procedures employed for both packing and cryopreservation had a significant impact on the viability of the DBs (Table 1). DBs that were unpacked, those stored in cryovials, and those enclosed in aluminum-foil bags demonstrated viability rates of 45.56%, 53.33%, and 66.67%, respectively, when subjected to the CP3 cryopreservation procedure. Additionally, when comparing twigs that were unpacked and those stored in cryovials, it was observed that the twigs vacuum sealed in the aluminum-foil bags exhibited superior bud viability (66.67%) across all three cryopreservation protocols (CP1, CP2, and CP3). In summary, twigs that were vacuum sealed in aluminum-foil bags and cryopreserved using the CP3 protocol exhibited the highest viability rates. Furthermore, twigs stored in cryovials and vacuum sealed in aluminum-foil bags demonstrated enhanced bud viability with the CP2 procedure in comparison to CP1; conversely, twigs that were unpacked showed greater bud viability with the CP1 procedure relative to CP2.

CP1: Samples were immersed in LN directly; CP2: Samples were subjected to a decreasing temperature gradient: −10 °C for 6 h, −20 °C for 6 h, and −30 °C for 6 h in a low temperature test chamber, followed by −80 °C in an ultra-low temperature freezer for 6 h, and finally immersed in LN; CP3: Samples were processed using a CryoMed controlled rate freezer, which decreased the temperature at a rate of 0.1 °C per min to −30 °C, maintaining this temperature for 24 h. This was followed by a temperature decline of 0.5 °C per min to −80 °C, then a decline of 1 °C per min to −180 °C, before being transferred to LN.

### 3.3. Effect of Thawing Procedures on the Viability of V. amurensis Buds

**Figure 5:**
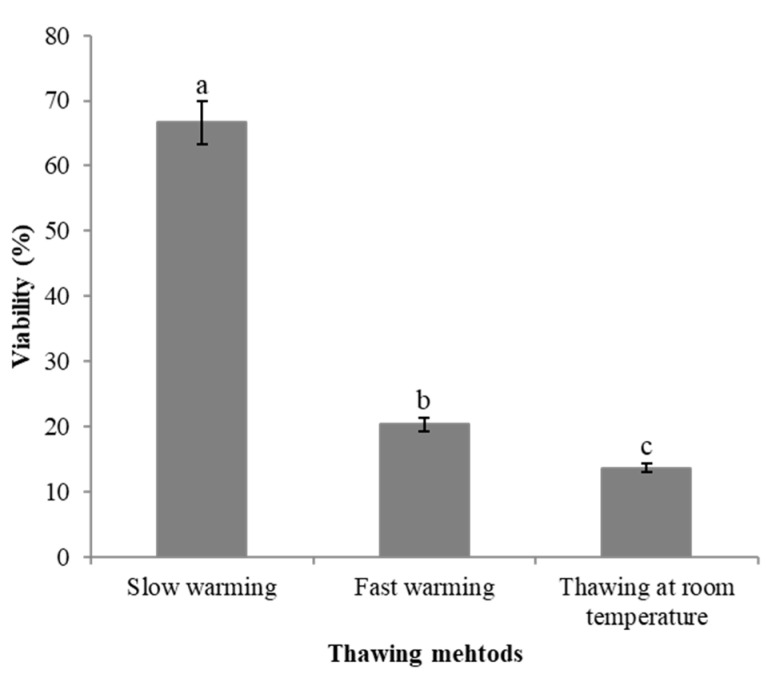
Effects of different thawing methods on the viability of dormant buds of ‘Shuanghong’ collected in January 2018 after cryopreservation. Note: Mean values with the same letter were not significantly different at *p* < 0.05 by the Duncan’s multiple range test.

The thawing method has a significant effect on the recovery of DBs. Specifically, DBs that underwent a 24 h slow thaw at 4 °C demonstrated a markedly higher viability rate of 66.67%. In contrast, the viability rates for fast thawing at 40 °C and at room temperature were only 20% and 13.33%, respectively (Figure 5). To improve water absorption and reduce the dissolution of pectin from the damaged cell walls resulting from cryopreservation, we implemented a protocol to change the water every hour during the initial 6 h of the slow thawing process.

### 3.4. Varieties and Harvest Time on the Viability of V. amurensis Buds

DBs harvested at different times exhibited significant variation in regeneration rates, ranging from 24.44% to 66.67% after cryopreservation. Buds collected from various genotypes at similar temperatures demonstrated considerable differences in their freezing tolerance. Notably, DB from all three varieties collected in December, January, and February displayed the highest viability following cryopreservation: ‘Shuanghong’ (55.56%, 66.67%, and 61.11%, respectively), ‘Zuoshanyi’ (55.56%, 60.00%, and 58.89%, respectively), and ‘Shuangfeng’ (56.67%, 65.56%, and 62.22%, respectively) (Table 2). We hypothesize that the DBs on twigs harvested in November were insufficiently dormant to endure cryopreservation, while those collected in mid-March had completed their dormant phase and therefore could not survive the ultra-low temperatures of LN. The average temperatures at which the twigs were harvested showed no correlation with the viability of the cryopreserved DBs. Although the viability of the cryopreserved DBs was higher in January, the average temperature in January was actually higher than that in December. Consequently, it is advised that twigs are harvested only after sufficient cold hardiness has developed. The viability of *V. amurensis* DBs after cryopreservation between 2019 and 2021 was also examined, and the average viability of all exceeded 60% (Table 3).

### 3.5. Effect of Cryopreservation on Cell Ultrastructure

**Figure 6:**
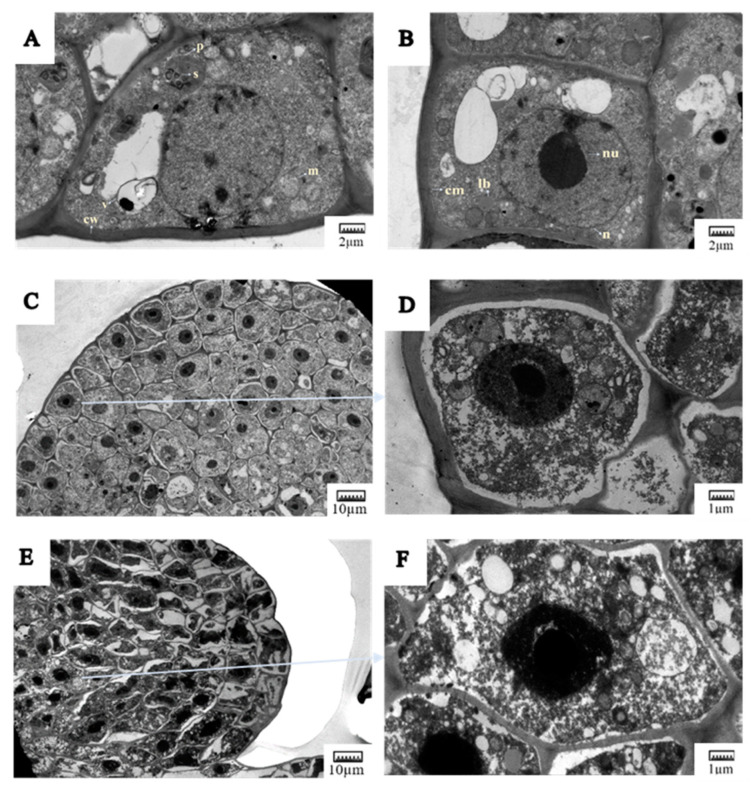
Ultrastructural observations of meristem cells in *V. amurensis* buds. (**A**,**B**) The buds before desiccation; (**C**,**D**) the buds desiccated for 28 d; (**E**,**F**) the buds after cryopreservation. Abbreviations: cm: cell membrane; cw: cell wall; lb: lipid body; m: mitochondrion; n: nucleus; nu: nucleolus; p: proplastid; s: starch; v: vacuole.

Transmission electron microscopy (TEM) was utilized to investigate the effects of desiccation and cryopreservation on meristematic cells in axillary buds. The non-cryopreserved (control) DBs exhibited large, prominent nuclei, numerous clear starch granules within plastids, and an abundance of mitochondria. These buds also displayed an intact nuclear membrane, a thick cell wall, and a complete nuclear skeleton (Figure 6A,B). Furthermore, the cell wall and cell membrane were in close proximity, with the cell membrane appearing smooth, orderly, and maintaining a distinct bilayer structure in the control buds. Dehydration removes free water from cells, thereby enhancing the stability of the cell membrane and other cellular structures. Consistently, dehydration resulted in an increase in cytoplasmic thickness and the coloration of the nuclei and nucleoli, a reduction in the abundance of large vacuoles, improved visibility of the mitochondria, and a slight separation between the cell wall and cell membrane. Nevertheless, the overall structure remained intact following dehydration (Figure 6C,D).

After cryopreservation, the outer six multilayered cells sustained irreparable damage, resulting in significant alterations to their structure (Figure 6E,F). In the cryopreserved samples, the gap between the cell wall and the cell membrane increased, causing the cytoplasm and nucleus to be compressed together. The plasmodesmata were disrupted, and the organelles appeared indistinct. Some cells exhibited large vacuole-like cavities filled with fibrous substances, likely representing debris formed by the disintegration of certain organelles due to drastic temperature changes, which led to lethal damage to the cells. Furthermore, the plasma membranes were compromised to varying extents, leading to an uneven surface or complete rupture. Starch, which is associated with protection against freezing injury, was not detected in the plastids. Despite some structural changes and slight plasmolysis, the endothelial cells remained intact and contained dark-colored nuclei. The endothelial cells demonstrated the capacity to resume growth following thawing, as the damage incurred during cryopreservation was assessed to be moderate. The following experiments also demonstrated that the DBs after cryopreservation had the ability to sprout after grafting (Figure 6).

## 4. Discussion

Cryopreservation protocols have been described with various propagules for grapevines such as pollen [41,42], seeds [43], somatic embryos [44,45,46,47,48], and shoot tips [35,36,37]. The method of cryopreservation for dormant buds is gaining significant interest for the preservation of genetic resources in woody plants because this method is easy to operate, and the technical requirements are not particularly high. Buds intended for cryopreservation can be collected from either field-grown or greenhouse plants of the tested genotypes. Furthermore, this technique has emerged as a viable option for cryopreservation that does not require in vitro laboratory support. In 1960, Sakai demonstrated the viability of dormant winter twigs from *Salix* and *Populus* under low-temperature conditions by slowly freezing the twigs at −30 °C before immersing them in LN [16]. Among the cryopreservation protocols developed for *Populus*, the cryostorage of dormant in vivo buds is the most suitable technique for ensuring a backup resource for field collections [49,50,51,52]. An efficient cryopreservation protocol for *V. amurensis* DBs was developed for the first time in this study.

The desiccation of twigs prior to cryopreservation requires considerable effort and time, and it is a widely used technique in many dormant bud cryopreservation protocols. Desiccation reduces the moisture content of DBs, which may decrease the cryodamage and enhance bud recovery. Dehydrating samples to a moisture content of 25–35% of their fresh weight before storage in LN has been shown to improve recovery success post-cryopreservation, depending on the species, compared to samples that were not dehydrated [53]. Vogiatzi et al. indicated that the key volume reduction in bulk water varied among apple varieties and that slow cooling under winter conditions to 30% had a significant effect on their survival [54]. According to Volk et al., *Malus* buds exhibited high viability after 10 years of storage in LN vapor, with some accessions demonstrating better viability when desiccated prior to slow cooling compared to those that were not desiccated [53]. During the cold acclimation process in winter, all apple varieties exhibited resistance to moderate dehydration stress. Höfer concluded that the initial moisture content and duration of dehydration had no impact on viability following cryopreservation, as all materials were desiccated to 30% moisture before exposure to LN [55]. However, opinions vary regarding the necessity of dehydration prior to cryopreservation. Some fruit trees do not require pre-freezing desiccation [56]. Esensee et al. reported that buds desiccated to 18% moisture content resulted in survival for certain ’Valiant’ grape buds [38]. For instance, Rantala et al. suggested that dormant blackcurrant buds should retain their natural moisture content to enhance the cryopreservation process [57]. In the present study, the moisture content of dormant *V. amurensis* buds was approximately 25–30%, which was beneficial for bud viability after cryopreservation. Excessive moisture may lead to the formation of large ice crystals that can damage cellular tissue, which is detrimental to the cryopreservation of *V. amurensis* buds.

Another important parameter is the cryopreservation protocol, which can ensure high recovery rates following cryopreservation. In this study, we investigated the twigs of *V. amurensis* using three types of packaging and three cryopreservation procedures. The results indicated that twigs packed in vacuum-sealed aluminum-foil bags exhibited greater viability across all three cryopreservation methods compared to the other two packaging types. Notably, the twigs packed in vacuum-sealed aluminum-foil bags and cryopreserved using the CP3 method achieved the highest viability. In the current study, the sections were desiccated to a moisture content of 25–30% in a refrigerator at −5 °C. Pre-freezing was conducted using a CryoMed controlled rate freezer, with a cooling rate of 0.1 °C·min^−1^ to −30 °C, followed by a 24-h hold at −30°C. The temperature was then decreased at a rate of 0.5 °C/min to −80 °C, and subsequently at 1 °C/min to −180 °C (CP3) in the same CryoMed controlled rate freezer (Forma 7451TF, Thermo Forma). After this process, the twigs were submerged in LN for 28 days. This procedure aligned with the three-step desiccation process utilized in *Malus* [58], which also indicates that desiccation to a 30% moisture content in a cold chamber at −5 °C is effective. The pre-freezing phase involved a controlled rate freezer with a cooling rate of 1 °C·h^−1^ to −30 °C. The 24-h preservation at −30 °C provided efficient cold hardening. To enhance recovery, the treatment of artificial cold acclimation following collection is likely to benefit the buds’ cold acclimation, thereby improving their resistance to cryopreservation. Sakai conducted classic research that identified a temperature range of −3 to −5 °C as the most effective for maximizing frost hardiness in twigs [59]. Vogiatzi et al. reported that the desiccation step at −4 °C was essential, and that the 24-h holding period at −30 °C contributed to improved recovery [13].

The distinct thawing methods can significantly influence the recovery of buds. In this study, slow thawing, which involved immersing the twigs of *V. amurensis* in water for 24 h at 4 °C in a refrigerator before transferring them to a substrate, resulted in markedly better recovery compared to those that were rapidly thawed. This difference may be attributed to the fact that slow thawing gradually restores the water absorption capacity of DBs, which lose excessive water during the desiccation process. Slow thawing has been widely utilized in *Malus* species. The cryopreserved tissues of *Malus* germplasm were subjected to gradual thawing through a nearly 24-h transfer to 4 °C in a cold room prior to removing the sections from vials and placing them in wet, autoclaved sand at the same temperature for 15 days of dehydration [38,58]. The researchers conducted a series of rehydration time tests on the preservation of different genotypes [55], and the results indicated that the genotypes exhibited varying requirements for thawing time. Therefore, the thawing duration for different genotypes of *V. amurensis* needs to be further adjusted and investigated.

Genotype and its response to the environment are equally important factors in cryopreservation. The genotype may influence the recovery of DBs following cryopreservation. Vogiatzi et al. demonstrated that the viability of winter-dormant apple buds after cryopreservation is strongly genotype-dependent [59]. Höfer conducted a detailed analysis of data from various *Malus* germplasms, indicating that the variability within a specific species deviated significantly from the standard deviations [60]. Genotypic differences in freezing tolerance were not reflected in the moisture content of the trees [61]. Reed showed that there was some genotypic variation in recovery; however, the viability of all genotypes exceeded the minimum threshold of 40% proposed as an acceptable limit for germplasm conservation [62]. Towill et al. adjusted the system to enhance the successful application of cryopreservation on dormant *Malus* buds, considering factors such as tolerance, moderation, and sensitivity [63]. In this study, the viabilities of the three species we harvested for cryopreservation all exceeded 55%. Therefore, the cryopreservation technique established in this research could be applied to the conservation of *V. amurensis* resources.

Dormancy, defined as the quiescent status of tissues and organs, may be relevant for cryopreservation due to its physiological basis related to growth systems and mechanisms of cold hardiness. Cold hardiness in woody perennials is influenced by a complex interplay of factors including the timing and rate of cold acclimation as well as the maximum level of cold tolerance achieved [64]. Furthermore, winter conditions prior to scion cutting significantly affect the moisture content and distribution within bud tissues, thereby impacting the recovery rate of DBs after cryopreservation [65]. Bilavcik et al. reported a direct correlation between cryosurvival and cold hardening [66]. The dormancy phase and extreme cold conditions exert notable effects on the post-cryopreservation survival of dormant *Malus* buds [13,39,56,59,67]. In this study, the viability of DBs of *V. amurensis* collected in November (autumn) and March (spring) was significantly lower compared to buds harvested in winter (January, February, and December). The majority of buds collected in November likely lacked sufficient dormancy to withstand cryopreservation. In contrast, buds collected in March had already exited their dormancy period, making them unable to survive the extremely low temperatures of LN. The duration of dormancy varies among different plant species across distinct regions. For dormant buds, cryopreservation is primarily suitable for species undergoing a dormancy phase, with low-temperature periods in the Northern Hemisphere typically occurring from late November to the end of January, occasionally extending into early February [68]. The timing for the successful cryopreservation of hybrid aspen aligns with findings that the maximum cold hardiness of forest trees in Finland is achieved between November and January [69]. Over 66% of DBs from *P. tremula*, originating from both coastal and continental Norway, have been shown to survive immersion in LN when collected in January and February [48]. In *P. tremuloides*, the cryopreservation of DBs collected from the mountains of Colorado, USA, demonstrated good survival rates of 79–92% in November and 100% in December [36]. Different from other grape varieties, *V. amurensis* is the most cold-resistant grape, so further research is needed to access the effectiveness of the proposed protocol on other grapevine species before its implementation.

The ultrastructure of cells in DBs of *V. amurensis* preserved at ultra-low temperatures was examined. It was observed that following the dehydration of meristematic cells, the degree of vacuolation decreased, along with a reduction in the content of free water within the cells. The ultrastructural changes in dehydrated cells serve as indicators of enhanced freeze resistance and dehydration tolerance, suggesting that these ultrastructural alterations are correlated with physiological changes, especially the water content and tissue [70]. Dehydration is a critical step in cryopreservation; only by removing sufficient water can cells achieve vitrification without the formation of ice crystals during rapid freezing. The cell membrane is a primary indicator of cellular damage during freezing, and electrolyte leakage serves as a measure of membrane integrity. Therefore, maintaining the integrity of the cell membrane is essential for maximizing the recovery rate of frozen samples. After cryopreservation, the perinuclear space expands, leading to significant nuclear deformation, severe contraction of the protoplasm, extensive damage to various organelles and membrane structures, and potentially lethal effects on the cells. Additionally, most of the cells experienced dehydration, which significantly enhanced their freeze resistance. Ultrastructural observations revealed that while the cell structure was slightly deformed, the organelles and membranes remained largely intact. Following thawing, the cells exhibited high viability, indicating that this cryopreservation method is effective for preserving the DBs of *V. amurensis*.

## 5. Conclusions

This is the first detailed report on the cryopreservation of DBs of *V. amurensis*, to the best of our knowledge. An improved recovery rate of 66.7% from the initial 30% was achieved through the program as follows. The bud viability was reached at a moisture content range of 25–30%. Then, the temperature of the twigs was decreased at a rate of 0.1 °C/min to −30 °C, maintaining this temperature for 24 h, followed by a decline of 0.5 °C/min to −80 °C, then a decline of 1 °C/min to −180 °C, before finally reaching −196 °C in a CryoMed controlled rate freezer. The low-temperature thawing procedure consisted of immersing the twigs in ice-cold (4 °C) water for 24 h and changing the water every hour during the initial 6 h to facilitate recovery. Twigs packed in aluminum-foil bags resulted in significantly higher recovery rates. Regarding the cell ultrastructure, the most significant changes in cells occurred during the processes of dehydration, freezing, and thawing. Therefore, optimizing the preservation procedure to achieve complete vitrification and enhance the relative viability of preserved cells is crucial for vitrification cryopreservation technology. In summary, it is only through a comprehensive investigation of the mechanisms underlying freezing injury and cellular antifreeze responses that we can establish a set of universally effective and straightforward cryopreservation methods for plant tissues and cells. This advancement will enable cryopreservation technology for plant cells to play a more significant role in bioengineering research and applications.

## Figures and Tables

**Table 1:** Effects of different packing modes and cryopreservation procedure on the viability of the dormant buds of ‘Shuanghong’ collected in January 2018 after cryopreservation.

Packing Mode	Moisture Content in Twigs (%)	Cryopreservation Procedure	Average Viability of Cryopreserved Buds (%) ± Standard Errors (SD)
No packing	28~30	CP1	30.00 ± 2 de
	28~30	CP2	25.56 ± 6 e
	28~30	CP3	45.56 ± 8 c
Cryovial	28~30	CP1	33.33 ± 2 d
	28~30	CP2	36.67 ± 2 d
	28~30	CP3	53.33 ± 4 b
Aluminum foil bag	28~30	CP1	42.22 ± 4 cd
	28~30	CP2	48.89 ± 5 c
	28~30	CP3	66.67 ± 4 a

Note: Mean values in each column with the same letter were not significantly different at *p* < 0.05 by the Duncan’s multiple range test.

**Table 2:** Effects of genotype and harvest time on the viability of *V. amurensis* dormant buds after cryopreservation.

Variety	Harvest Date (mm/dd/yy)	Total Moisture Content (%)	Total Moisture Content After 28 Days Desiccation (%)	Average Viability of CK (%)	Average Viability After Cryopreservation (%)
‘Shuanghong’	11/10/17	42.16	29.49	92.22 ± 4 b	37.78 ± 4 d
	12/10/17	40.33	28.78	97.78 ± 2 a	55.56 ± 8 c
	01/08/18	41.06	27.78	83.33 ± 8 c	66.67 ± 1 2 a
	02/01/18	45.10	28.03	87.78 ± 3 c	61.11 ± 6 b
	03/03/18	51.60	29.00	87.78 ± 4 c	36.67 ± 2 d
‘Zuoshanyi’	11/10/17	44.63	27.86	88.89 ± 4 b	26.67 ± 2 c
	12/10/17	44.47	28.30	93.33 ± 5 a	55.56 ± 9 a
	01/08/18	43.10	28.04	84.44 ± 1 2 b	60.00 ± 10 a
	02/01/18	43.25	27.98	92.22 ± 4 a	58.89 ± 3 a
	03/03/18	48.03	27.56	83.33 ± 5 b	37.78 ± 4 b
‘Shuangfeng’	11/10/17	46.61	28.74	87.78 ± 7 b	24.44 ± 4 c
	12/10/17	48.18	29.01	82.22 ± 5 b	56.67 ± 5 b
	01/08/18	46.61	28.36	88.89 ± 11 b	65.56 ± 7 a
	02/01/18	48.18	28.03	90.00 ± 5 a	62.22 ± 6 b
	03/03/18	52.74	27.98	88.89 ± 4 b	32.23 ± 4 c

Note: CK represents the twigs before cryopreservation. Mean values in each column with the same letter were not significantly different at *p* < 0.05 by the Duncan’s multiple range test.

**Table 3:** The viability of *V. amurensis* DBs after cryopreservation between 2019 and 2021.

Variety	Moisture Content in Twigs (%)	Average Viability in 2019 (%)	Average Viability in 2020 (%)	Average Viability in 2021 (%)
‘Shuanghong’	28–30%	68.89 ± 6 a	66.33 ± 9 a	70.00 ± 5 a
‘Zuoshanyi’	28–30%	62.11 ± 2 b	64.67 ± 8 b	63.11 ± 5 c
‘Shuangfeng’	28–30%	66.67 ± 2 a	66.12 ± 5 a	67.44 ± 3 b

Note: Mean values in each column with the same letter were not significantly different at *p* < 0.05 by the Duncan’s multiple range test.

## Data Availability

The authors will supply the relevant data in response to reasonable requests.
